# The role of cognitive flexibility on higher level executive functions in mild cognitive impairment and healthy older adults

**DOI:** 10.1186/s40359-024-01807-5

**Published:** 2024-05-30

**Authors:** Ilaria Corbo, Giovanna Troisi, Giulia Marselli, Maria Casagrande

**Affiliations:** 1 Department of Dynamic and Clinical Psychology, and Health Studies, “Sapienza” University of Rome, Via degli Apuli 1 - 00185, Rome, Italy; 2 Department of Psychology, “Sapienza” University of Rome, Rome, Italy

**Keywords:** Mild Cognitive Impairment, Aging, Executive Functions, Cognitive Flexibility, Planning, Fluid Intelligence

## Abstract

**Background:**

Mild Cognitive Impairment (MCI) is a preclinical condition between healthy and pathological aging, which is characterized by impairments in executive functions (EFs), including cognitive flexibility. According to Diamond’s model, cognitive flexibility is a core executive function, along with working memory and inhibition, but it requires the development of these last EFs to reach its full potential. In this model, planning and fluid intelligence are considered higher-level EFs. Given their central role in enabling individuals to adapt their daily life behavior efficiently, the goal is to gain valuable insight into the functionality of cognitive flexibility in a preclinical form of cognitive decline. This study aims to investigate the role of cognitive flexibility and its components, set-shifting and switching, in MCI. The hypotheses are as follows: (I) healthy participants are expected to perform better than those with MCI on cognitive flexibility and higher-level EFs tasks, taking into account the mediating role of global cognitive functioning; (II) cognitive flexibility can predict performance on higher-level EFs (i.e., planning and fluid intelligence) tasks differently in healthy individuals and those diagnosed with MCI.

**Methods:**

Ninety participants were selected and divided into a healthy control group (*N* = 45; mean age 64.1 ± 6.80; 66.6% female) and an MCI group (*N* = 45; mean age 65.2 ± 8.14; 40% female). Cognitive flexibility, fluid intelligence, planning, and global cognitive functioning of all participants were assessed using standardized tasks.

**Results:**

Results indicated that individuals with MCI showed greater impairment in global cognitive functioning and EFs performance. Furthermore, the study confirms the predictive role of cognitive flexibility for higher EFs in individuals with MCI and only partially in healthy older adults.

## Background

Mild Cognitive Impairment (MCI) is a preclinical condition that lies between healthy and pathological aging. According to Petersen’s criteria [1], a diagnosis of MCI involves (I) one or more cognitive deficits that are worse than expected for the person’s age and education, (II) preserved abilities to perform daily life activities, and (III) not meeting the criteria for a diagnosis of dementia. MCI is classified as either amnestic (aMCI) or non-amnestic (naMCI), depending on whether memory is affected or not. In addition, MCI can be further classified based on the number of cognitive domains impaired, with single-domain MCI (i.e., aMCI and naMCI) or multiple-domain MCI (i.e., aMCI-md, naMCI-md) [2]. Mild Cognitive Impairment is associated with various psychological, physiological, and cognitive symptoms [3–19]. The most common cognitive symptoms are deficits in executive functions (EFs) [5, 7, 8], which are higher-level cognitive functions that allow individuals to adapt their behavior by integrating internal and environmental stimuli [20].

Diamond’s model [20] is the most widely accepted theoretical framework of executive functioning. The proposed structure is hierarchical and includes three core executive functions: working memory, inhibitory control, and cognitive flexibility. However, cognitive flexibility requires the development of working memory and inhibitory control to reach its full potential. These EFs support higher-level EFs, such as planning and fluid intelligence. Working memory is the process of holding and manipulating information in the mind; inhibitory control is the ability to manage attention, behavior, thoughts, and emotions, enabling individuals to not be slaves to habits and automaticity. Cognitive flexibility develops later than other core EFs as well [20]. It is considered a core executive function that enables individuals to adapt their behavior in response to unexpected and novel environmental demands. Its importance lies in facilitating appropriate behaviors and efficient work by allowing us to disengage from the current task and approach a new one [21, 22]. In doing so, it requires the ability to inhibit previous perspectives and manipulate environmental information through working memory. Despite being poorly understood, this ability has been described in various ways over the years [22, 23].

Cognitive flexibility is the ability to switch between multiple tasks or mental sets (set-shifting), to perform a series of tasks sequentially by stopping the current task and switching to the next one (task-switching), to change perspectives by activating or inhibiting different alternatives, to solve complex problems. It also involves creative thinking, action planning, multitasking, categorization, and flexible use of language. Cognitive flexibility also appears to be involved in psychological aspects such as coping with anxiety, stress, and anger. It is important to note that cognitive flexibility requires other core executive functions to fulfill its functionality [20, 22–27] These different interpretations of flexible behavior not only contribute to the complexity of the construct but also create challenges in assessing it in an experimental context.

A recent review [28] analyzed the most commonly used neuropsychological tests to assess this ability in healthy individuals. The review identified the Trail Making Test (TMT), Wisconsin Card Sorting Test (WCST), and Controlled Oral Word Association Test (COWA) as the most widely used tasks. The Trail Making Test and the Wisconsin Card Sorting Test are typically used to assess set-shifting [20, 29–32]. The COWA is commonly used to assess verbal fluency and switching [33, 34]. This work highlights the complexity of this construct, which has several facets. The analysis of cognitive flexibility has been approached in two ways: as a single unique construct (e.g [20, 21]), or by examining individual components, such as set-shifting or task switching (e.g [25, 30]). However, it is important to consider how these different components may act simultaneously and interact with each other.

From core EFs, develop the higher-level EFs: planning and fluid intelligence (gF). Fluid intelligence integrates reasoning and problem-solving skills and enables individuals to deal with novel situations by identifying and manipulating the available information to arrive at a solution [35]. Planning involves the ability to organize behavior and anticipate a goal through a sequence of intermediate steps. The process consists of two levels: the plan formulation and the execution of that plan. The plan formulation requires the development of a logical approach to achieve the established goal, while the execution plan enables close monitoring of activities to achieve the goal [36]. Fluid intelligence and planning are both higher-level executive functions that require the presence of other executive functions in order to develop appropriately and be functional for the individual; therefore, both are closely related to basic EFs, including cognitive flexibility [20].

Executive functions remain stable throughout adulthood and gradually decline with age [37], as a consequence of their dependence on prefrontal lobe functioning [38], particularly the prefrontal cortex (PFC), which is the region most affected by physiological aging [39]. These changes can lead to a decline in cognitive flexibility, planning, and fluid intelligence tasks in healthy individuals [37, 39–41]. This deterioration is even more evident in pathological forms of aging, such as MCI. Several systematic reviews have addressed this executive impairment in MCI (i.e [5, 7, 8]). Individuals with MCI frequently exhibit impairments in various cognitive domains, including switching, set-shifting, inhibitory control, working memory, planning, reasoning, fluid intelligence, problem-solving, cognitive flexibility, and abstract thinking [5, 7, 8, 42]. Although a dysexecutive syndrome is frequently found in MCI, to our knowledge, no study has simultaneously analyzed the different components of cognitive flexibility, and only a few studies have focused on the role of core executive functions on higher-level executive functioning. For example, Zhang and colleagues found differences in planning abilities between healthy older adults and older individuals with MCI in the absence of significant differences in inhibitory control.

Given the central role of cognitive flexibility in the successful performance of higher-level EFs and its specific role for higher-level EFs, a more comprehensive examination of its role in healthy and preclinical aging could provide useful insights.

## Methods

### Aims

Given the variety of cognitive deficits present in Mild Cognitive Impairment and especially in executive functioning [7, 8], it is hypothesized that I) healthy subjects will perform better than participants with Mild Cognitive Impairment on cognitive flexibility and higher-level executive functioning. It is further hypothesized that global cognitive functioning (MMSE score) will play a mediating role. Moreover, due to the lack of studies examining (a) the components of cognitive flexibility, (b) the relationship between cognitive flexibility (and its components) and higher-level executive functioning in different forms of aging; given the central role of cognitive flexibility in higher-level executive functioning [20] and the role of switching and set-shifting in defining cognitive flexibility [22, 43], the second hypothesis is that II) the components of cognitive flexibility can predict higher-level executive functions in older adults with Mild Cognitive Impairment, with the mediating role of global cognitive functioning, as measured by the MMSE score.

### Participants

Ninety participants aged 50 years and older took part in the study (age range: 50–81 years, mean age 64.7 ± 7.48). The total sample was divided into two groups according to the diagnosis of Mild Cognitive Impairment: healthy (HC) and MCI. The healthy control group (*N* = 45; age range: 51–75) had a mean age of 64.1 ± 6.80 and was 66.6% (30 of 45) female. The group with MCI (*N* = 45; age range: 50–81) had a mean age of 65.2 ± 8.14 and was composed of 71% (32 of 45) females. In addition, the group with MCI consisted of 40% (18 of 45) of participants with aMCI. The following exclusion criteria were adopted: neurological disorders (e.g., stroke, epilepsy, Parkinson’s disease, dementia), psychiatric disorders (e.g., bipolar disorder, schizophrenia), the presence of chronic medical conditions (e.g., head trauma, brain injury), color blindness; Mini Mental State Examination ≤ 23 [44]. Therefore, we excluded participants with medical conditions that could influence cognitive functions, in order to control for any potential comorbidity that could interfere with the study of mild cognitive impairment. An independent sample of 300 healthy participants (see [43]) was employed to obtain the factor weights with the purpose of developing a weighted composite score of switching and set-shifting.

### Diagnosis of mild cognitive impairment

Mild Cognitive Impairment was diagnosed according to the most common standard criteria [1, 45, 46]. Participants were classified as having “MCI” if they displayed impairment in at least one cognitive domain and had a score below the threshold of 1.5 standard deviations with respect to the reference sample. Neuropsychological tests used for diagnosis assessed several cognitive domains: memory, visuospatial skills, language, executive functioning, and attention.

### Measures

#### Socio-demographic and anamnestic information

Demographic data (i.e., age, sex, and years of education), medical history, familiarity for certain pathologies (e.g., cardiovascular diseases, dementia), and psychiatric information were collected for each patient by a face-to-face interview.

#### Neuropsychological assessment used for the diagnosis of mild cognitive impairment

##### Memory

*Digit Span Forward* [47].

The task is used to assess verbal short-term memory; the participant is asked to repeat after the experimenter a series of digits in the same order in which they were pronounced. if the participant repeats the string correctly, the experimenter will pronounce another string containing one more digit than the previous one; if the participant fails, another string with the same number of digits will be administered. The test ends after two consecutive failures. The score obtained corresponds to the short-term memory span.

*Rey Auditory Verbal Learning Test* [48].

It assesses immediate and delayed memory. The test consists of 5 repetitions of a list of 15 words, each followed by the participant’s immediate recall. Delayed recall is carried out once after 15 min, during which the participant is engaged in visual-spatial tasks. A score of 1 is assigned for each recalled word in every single repetition; thus, the total immediate score ranges from 0 to 75, while the maximum total delayed score is 15.

*Babcock’s Story Recall Test* [49].

It assesses short- and long-term semantic memory. The experimenter reads a short story that the subject is asked to repeat immediately. After that, the experimenter reads the story for the last time and asks the participant to recall it later, specifically after 10 min, during which the participant is engaged in visual-spatial tasks. A maximum score of 8 for each recall is assigned according to the main events of the story and the details recalled by the participants.

*Immediate Visual Memory Test* [50].

It assesses short-term visuospatial memory. A figure stimulus is presented for 3 s, and the participant is asked to identify it among four alternatives. The test consists of 21 stimuli; a score of 1 is given for each correct response, with a total score ranging from 0 to 21.

##### Visuospatial abilities

*Copying Drawings With and Without Programming Elements* [50].

It assesses the praxis and visuospatial abilities of the individual. The first part of the test consists of copying the proposed draw (stimulus); other trials containing some graphic features of the original stimulus, such as lines, dots, or angles, are shown; the participant is asked to complete the missing parts and reproduce the figure-stimulus. A point is assigned for each missing part completed correctly.

*Clock Drawing Test* [51].

It assesses visuospatial abilities. A circle drawn on a blank page is presented; the participant is required to insert the numbers of the clock within the circle. Then, the participant is asked to draw the hands of the clock indicating a specific time (11:10).

*Rey-Osterrieth Complex Figure Test* [52].

It assesses constructive praxis and long-term visuospatial memory. The participant is asked to reproduce a complex line drawing, copying it freehand (copy), and then drawing it from memory (recall) after 10 min. The final score depends on the accuracy (position and reproduction) of the elements copied and recalled.

*Sentence Construction Test* [50].

It assesses the ability to produce sentences from a set of words provided by the examiner. The test consists of 5 items. Each correct sentence scores 3 when correct, and 1 or 2 more points if it was constructed in less than 20–10 s respectively.

##### Attention

*Visual Search* [53].

It assesses selective attention. Participants must determine the presence or absence of the target stimulus by detecting it among a set of stimulus distractors. It consists of three different matrices of digits with increasing attentional load; the participant must mark off the target stimulus/i (e.g., a digit in the first matrix, two digits in the second one, and three digits for the third). The target stimuli are 10 in the first matrix, 20 in the second, and 30 in the third. The final score is given by the number of digits identified within a time of 45 s.

*Trail Making Test A (TMT A;* [54]*)*.

TMT A assesses attention and processing speed. In this part of the test, the participant must join – in increasing order – a set of numbers [1–13] randomly arranged on a sheet in the shortest time possible. The score is given by the number of seconds taken to complete the task.

##### Executive functions

*Digit Span Backward* [47].

This test assesses working memory. Similarly to the Digit Span Forward, the experimenter reads different strings of digits, one at once; the participant is required to repeat the string of digits in the same order but backward. If the participant repeats the sequence in the right order, a string with a digit more is read by the experimenter; if the participant fails, another string with the same number of digits is read. The test ends after two consecutive failures. The score obtained corresponds to the span of working memory.

##### Global cognitive functioning

*Mini-Mental State Examination (MMSE;* [55]*)*.

The MMSE assesses global cognitive functioning by measuring temporal orientation, spatial orientation, short-term memory, computation, attention, recall memory, language, and praxis skills. The maximum score obtainable is 30; a score equal to or lower than 23 indicates the presence of cognitive impairment ranging from mild to severe [Tombaugh and McIntyre, 1992].

*Activities of Daily Living (ADL;* [56]*)*.

This questionnaire assesses the ability to perform activities of daily living. It consists of 6 items that assess a person’s independence in personal hygiene, mobility, nutrition, and continence. The score ranges from 0 (complete dependence) to 6 (autonomy in all functions).

*Instrumental Activities of Daily Living (IADL;* [57]*)*.

This questionnaire assesses the ability to perform activities necessary to maintain independence. It includes eight items that investigate the person’s functional independence in complex daily activities, such as using the telephone, shopping, preparing meals, taking care of the home, doing laundry, using transportation and the telephone, and managing therapy and money. The score ranges from 0 (complete dependence) to 8 (independence in all functions).

##### Executive functions

###### Cognitive flexibility (set-shifting)

*Trail Making Test (TMT;* [54]*)*.

The TMT consists of two subtests, A and B. TMT B assesses set-shifting, and in this part, the participant must join numbers [1–13] and letters (A-N) alternately in the shortest time possible. The score is given by the number of seconds taken to complete the test.

*Computerized version - Wisconsin Card Sorting Test (WCST;* [58]*)*.

The WCST assesses set-shifting. The test requires that participants match cards according to the specific characteristics of four stimulus cards. The cards contain one of four possible symbols (stars, crosses, circles, triangles), a number from 1 to 4, and one of the four colors (red, yellow, green, blue). The four stimulus cards represent one red triangle, two green stars, three yellow crosses, and four blue circles. The participant is given two decks of 64 cards and must place a single card from the deck under one of the four stimulus cards. The participant is not informed about the criteria for matching the cards. After each choice made by the participant, the task provides feedback on the correctness of the response to allow the participant to deduce the criterion adopted and to sort the cards based on this feedback, ignoring the other criteria. The criterion adopted changes for every ten consecutive correct responses (color, shape, number, color, shape, number). The procedure continues until the two decks of cards or six sequences of correct answers are completed. Several indices can be extrapolated from the test (e.g., global score, perseverative errors, no perseverative errors, categorical failures), but in this study, we will examine only “perseverative errors”, which are the most indicative indices of set-shifting performance [59]. Perseverative errors are defined as all responses made following the previous category.

##### Cognitive flexibility (task switching)

*Phonemic Fluency Test (PF;* [60]*)*.

This test assesses phonemic verbal fluency, clustering, and task switching. The participants are required to say as many words as possible in one minute; words have to begin with a certain letter given by the experimenter. Three letters (i.e., L, F, P) are tested, so that the participant has three minutes to complete the test. The score is given by the total number of words produced by the participant, excluding any repetitions and proper names of persons and places.

*Semantic Fluency Test (SF;* [60]*)*.

This test assesses semantic verbal fluency skills, clustering, and task switching. The participant is required to say as many words as possible that belong to a given semantic category. Three categories (fruits, animals, car brands) are tested, and the participant has to say the words in a minute for each category. The score is given by the total number of words produced by the participant, excluding any repetitions.

##### Planning

*Tower of London (ToL;* [61]*)*.

ToL is a test designed to assess planning and problem-solving skills; it consists of a base with three pegs of different lengths and three colored spheres (red, blue, and green). The test consists of 16 problems in which one must mentally plan a sequence of limited moves to reproduce a given configuration/arrangement of the spheres. Each configuration is scored from 3 to 0, based on the number of attempts made to solve the test. Several indices (e.g., total score, decision time, execution time, number of rule violations) can be extrapolated from the test, but in this study, we will only examine the global score, which is given by the sum of the score obtained on each trial (0–48).

##### Fluid Intelligence

*Raven’s Standard Progressive Matrices (RSPM;* [62]*)*.

RPM is a test of logical-deductive intelligence that assesses fluid intelligence. It consists of 60 tables, divided into five sets of 12 tables of increasing difficulty. Each table presents a stimulus figure characterized by the absence of a fragment and six to eight response alternatives. The participant must choose the alternative that better fulfills the picture from the proposed options. The score is given by the sum of the correct answers (0–60).

##### Procedure

Participants were recruited voluntarily through widespread advertising (posters, web ads, word of mouth). The purpose of the study was explained to each participant so they could voluntarily decide to participate or withdraw their participation. Informed consent to participate in this study was obtained from all participants. Then, participants underwent a face-to-face interview to determine their eligibility for participation in the study and, if applicable, reasons for exclusion. Data collected included socio-demographic and anamnestic information. Once accepted for participation, all individuals were administered a neuropsychological battery in a randomized order in order to determine Mild Cognitive Impairment; then, tests were administered to assess cognitive flexibility (Wisconsin Card Sorting Test, Trail Making Test, Phonemic and Semantic Fluency Tests), fluid intelligence (Raven’s Standard Progressive Matrices) and planning (Tower of London). The administration lasted about 4 h, divided into two parts, separated by an interval of about 20 min, or divided into two different days. In the first session, tests were administered to assess cognitive flexibility, fluid intelligence, and planning. In the second session, the neuropsychological battery was administered to diagnose Mild Cognitive Impairment. The entire procedure conformed to ethical standards and the Helsinki declaration for research involving human subjects. The Institutional Review Board of the Department of Dynamic and Clinical Psychology and Health Studies - Sapienza University of Rome approved the study (protocol number: 0000684).

##### Statistical analysis

Mild Cognitive Impairment was diagnosed using a cut-off of 1.5 standard deviations below the mean of normative data [1, 46]. To assess the first hypothesis, that is, to observe what differences there were between the healthy control (HC) and the participants with Mild Cognitive Impairment, a series of ANOVAs were conducted to assess differences in socio-demographic variables, the Mini Mental State Examination, the Instrumental Activities of Daily Living and the chi-square test (χ2) were used to assess differences in categorical variables. A MANCOVA was conducted to assess differences in cognitive flexibility and higher-level executive functions, considering the Group (HC or MCI) as the independent variable and executive functioning measures as the dependent variables; the Mini Mental State-Examination score was used as the covariate. ANCOVAs were performed to determine the contribution of each variable to the results of the MANCOVAs.

To assess the second hypothesis, the results of a previous confirmatory factor analysis (CFA) [43] were used to compute a weighted composite score for Switching (standardized score of the Phonemic Fluency Test and standardized score of the Semantic Fluency Test) and Set-Shifting (standardized score of the Perseverative Errors of Wisconsin Card Sorting Test and standardized score of the Trail Making Test B). The composite scores of the Switching and Set-shifting factors were calculated by summing the tests that saturated into factors 1 and 2 and multiplying by their factorial weights according to the following formulas:

$${\bf{Switching}} = {\rm{ }}\left( {0.78*{\rm{Z}}\_{\rm{PF}}} \right){\rm{ }} + {\rm{ }}\left( {0.63*{\rm{Z}}\_{\rm{SF}}} \right)$$

$${\bf{Set}} - {\bf{Shifting}} = {\rm{ }}\left[ {\left( {0.62*{\rm{Z}}\_{\rm{PE}}} \right){\rm{ }} + {\rm{ }}\left( {0.73*{\rm{Z}}\_{\rm{TMTB}}} \right)} \right]{\rm{ }}*\left( {{\rm{ }} - 1} \right)$$

To assess whether cognitive flexibility can predict higher executive functioning in aging with Mild Cognitive Impairment, linear regressions were performed on the total sample, the healthy older adults, and the older adults with MCI. In the linear regressions, planning and fluid intelligence were used as dependent variables. The standardized score of the Mini Mental State Examination, composite scores of switching, and set-shifting were used as independent variables. Linear regressions were performed on the global sample and separately for each group of participants (HC and MCI). Statistical analyses were performed using Jamovi (2.4.11).

## Results

### Socio-demographic and global functioning characteristics in healthy older adults and older adults with MCI

The results of the socio-demographic variables and the raw scores of the cognitive tests are shown in Table 1.

**Table 1:** Socio-demographic and global functioning characteristics in healthy older adults and older adults with MCI

	HC	MCI	χ ^2^ / F	*p*	η²*p*
Age range	51–75	50–81			
N°	45	45			
Age (Mean ± SD)	64.1 (6.80)	65.2 (8.14)	< 1	0.48	0.01
Sex (%F)	66.6	71	< 1	0.83	
Education (Mean ± SD)	15.7 (3.69)	14.6 (3.43)	1.94	0.17	0.02
Mini Mental State Examination (Mean ± SD)	29.4 (0.80)	28.5 (1.50)	12.3	**< 0.001**	0.12
Activities of Daily Living (Mean ± SD)	5.95 (0.21)	6 (0)	1.72	0.19	0.02
Instrumental Activities of Daily Living (Mean ± SD)	7.86 (0.41)	7.75 (0.81)	< 1	0.42	0.01

HC: Healthy Control; MCI: Mild Cognitive Impairment

### Comparison of healthy older adults (HC) and older adults with mild cognitive impairment in executive functions

#### Cognitive flexibility

The MANCOVA considered Group (HC vs. MCI) as the independent variable, Mini Mental State Examination score as the covariate, and raw scores of tests assessing cognitive flexibility (Phonemic Fluency Test, Semantic Fluency Test, Perseverative Errors - Wisconsin Card Sorting Test, Trail Making Test B) as the dependent variables showed a significant effect (F(4,79) = 5.27, Wilks’ = 0.79, *p* < 0.001) and ANCOVAs on each dependent variable indicated a significant role for Perseverative Errors - Wisconsin Card Sorting Test (F(4,79) = 12.42, *p* < 0.001) and Trail Making Test B score (F(4,79) = 15.69, *p* < 0.001; Table 2).

**Table 2:** Means (± SD) of cognitive flexibility in healthy and MCI adults and ANCOVA results

	HC	MCI	F	*p*	η²*p*
Phonemic Fluency Test	48.7 (11.40)	45.7 (12.09)	1.70	0.20	0.02
Semantic Fluency Test	53.1 (11.45)	51.9 (11.17)	< 1	0.38	0.01
Perseverative Errors	13.2 (9.75)	16.6 (13.21)	12.42	**< 0.001**	0.13
Trail Making Test B	75.8 (18.96)	110.3 (66.7)	15.69	**< 0.001**	0.19

HC: Healthy Control; MCI: Mild Cognitive Impairment

#### Higher-Level Executive functions

The MANCOVA considered the Group (HC vs. MCI) as independent variable, the Mini Mental State Examination score as covariate, and the raw scores of tests assessing higher executive functioning (Tower of London, Raven’s Standard Progressive Matrices) as the dependent variables showed a significant effect (F(6,91) = 6.49, Wilks’ = 0.87, *p* = 0.002) and ANCOVAs on the individual dependent variables indicated a significant role of both Raven’s Standard Progressive Matrices (F(2,83) = 12.34, *p* < 0.001) and Tower of London (F(2,83) = 6.07, *p* = 0.016; Table 3).

**Table 3:** Means (± SD) of higher-level executive functions in healthy and MCI adults and ANCOVA results

	HC	MCI	F	*p*	η²*p*
Tower of London	37.9 (3.89)	36.9 (4.53)	6.07	**0.016**	0.07
Raven’s Standard Progressive Matrices	42.8 (8.28)	35.7 (9.47)	12.34	**< 0.001**	0.13

HC: Healthy Control; MCI: Mild Cognitive Impairment

#### Linear regression

##### Total sample

###### Fluid Intelligence

Fluid intelligence (standardized Raven’s Standard Matrices test score) was considered as the dependent variable, and the standardized score of the Mini Mental State Examination, Switching, and Set-Shifting composite scores as predictors. The results show that the model is significant (R^2^ = 0.42, *p* < 0.001), and set-shifting is the variable that significantly predicts fluid intelligence (Table 4).

###### Planning

Planning (standardized score of the Tower of London) was considered as the dependent variable, and the standardized score of the Mini Mental State Examination, Switching, and Set-Shifting composite scores were used as predictors. The results have shown that the model is significant (R^2^ = 0.18, *p* = 0.002), and the set-shifting is the variable that significantly predicts planning (Table 4).

**Table 4:** Linear regression conducted on the total sample

	Fluid Intelligence			Planning		
	B	SE	*p*	B	SE	*p*
MMSE	0.17	0.10	0.113	0.08	0.13	0.553
Switching	0.07	0.08	0.351	-0.02	0.09	0.805
Set-Shifting	0.56	0.12	**< 0.001**	0.42	0.15	**0.006**
R	0.65			0.41		
R^2^	0.42			0.18		
p	**< 0.001**			**0.002**		

MMSE: Mini Mental State Examination

#### Healthy older adults

##### Fluid intelligence

Fluid intelligence (standardized Raven’s Standard Matrices test score) was considered as the dependent variable, and the standardized score of the Mini Mental State Examination, Switching, and Set-Shifting composite scores were used as predictors. The results revealed that the model is significant (R^2^ = 0.40, *p* < 0.001), and set-shifting is the variable that significantly predicts fluid intelligence (Table 5).

##### Planning

Planning (standardized score of the Tower of London) was considered as the dependent variable, and the standardized score of Mini Mental State Examination, Switching, and Set-Shifting composite scores were used as predictors. The results have shown that the model is not significant (*p* > 0.05), and cognitive flexibility does not predict planning in healthy older groups (Table 5).

**Table 5:** Linear regression performed on the healthy older group

	Fluid Intelligence			Planning		
	B	SE	*p*	B	SE	*p*
MMSE	0.30	0.18	0.099	0.18	0.23	0.432
Switching	0.06	0.11	0.554	0.10	0.14	0.488
Set-Shifting	0.70	0.19	**< 0.001**	0.15	0.26	0.566
R	0.64			0.25		
R^2^	0.40			0.06		
p	**< 0.001**			0.458		

MMSE: Mini Mental State Examination

#### Mild cognitive impairment

##### Fluid Intelligence

Fluid intelligence (standardized Raven’s Standard Matrices test score) was considered as the dependent variable, and the standardized score of Mini Mental State Examination, Switching, and Set-Shifting composite scores were used as predictors. The results have shown that the model is significant (R^2^ = 0.37, *p* < 0.001), and set-shifting is the variable that significantly predicts fluid intelligence (Table 6).

##### Planning

Planning (standardized score of the Tower of London) was considered as the dependent variable, and the standardized score of Mini Mental State Examination, Switching, and Set-Shifting composite scores as predictors. The results revealed that the model is significant (R^2 ^= 0.27, *p* = 0.008), and set-shifting is the variable that significantly predicts planning (Table 6).

**Table 6:** Linear regression conducted on the group with mild cognitive impairment

	Fluid Intelligence			Planning		
	B	SE	*p*	B	SE	*p*
MMSE	0.07	0.14	0.638	0.01	0.16	0.943
Switching	0.06	0.11	0.601	-0.10	0.13	0.432
Set-Shifting	0.49	0.16	**0.004**	0.55	0.19	**0.005**
R	0.61			0.52		
R^2^	0.37			0.27		
p	**< 0.001**			**0.008**		

MMSE: Mini Mental State Examination

## Discussion

This study aimed to analyze differences in higher-level executive functioning between healthy older adults and those with Mild Cognitive Impairment. The role of flexibility components, specifically set-shifting and task-switching, in planning and fluid intelligence was also analyzed.

Consistent with the first hypothesis, participants with Mild Cognitive Impairment exhibited poor executive functioning performance compared to healthy older adults. This relationship was found to be mediated by global cognitive functioning, as indexed by MMSE scores.

Executive functions remain stable throughout adulthood but progressively decline with age [37]. These functions rely on the functions of the frontal lobes [38], particularly the prefrontal cortices (PFCs), which are the brain regions most vulnerable to physiological aging [39]. The PFC undergoes an atrophic process starting at the ages 50–70, which leads to a decrease in cortical volume and a reduction in dopaminergic neurons [39]. These cerebral changes lead to a decline in cognitive abilities during physiological aging conditions [37, 39, 40]. This decline is more pronounced in conditions of preclinical and clinical forms of pathological aging (i.e [7, 8]). These cerebral changes account for the poorer performance of participants with MCI in executive functioning.

The results found that individuals with Mild Cognitive Impairment exhibit greater difficulties in both global functioning, as assessed by the Mini Mental State Examination, and executive functioning. Specifically, participants with MCI have greater impairment in one component of cognitive flexibility, namely set-shifting, as evidenced by their poor performance on the Trail Making Test B and the greater presence of perseveration errors on the Wisconsin Card Sorting Test. This finding could be explained by the multiple changes in the central nervous system that characterize Mild Cognitive Impairment.

Individuals with Mild Cognitive Impairment have more frequent structural alterations in various brain regions, including the pulvinar, middle frontal gyrus, temporal neocortex, superior temporal gyrus, frontal regions, ventrolateral and ventroposterior thalamus, parahippocampal gyrus, cingulate cortex, entorhinal cortex, and amygdala when compared to healthy older adults [63–67]. The ventrolateral thalamus is closely associated with striatal regions, which are involved in executive functioning [67]. Regarding set-shifting, older adults with MCI were found to have hypoperfusion in the anterior cingulate, striatum, and thalamus areas during the administration of the Trail Making Test B [66]. These structural and metabolic changes resulted in poorer performance on tasks assessing higher executive functioning, such as the Tower of London and Raven’s Standard Progressive Matrices. This finding can be explained by hypometabolism in the middle frontal gyrus during the administration of Raven’s Standard Progressive Matrices [65].

Executive functioning is a primary marker of cognitive decline, and its impairment can increase the risk of conversion between MCI and AD [68, 69]. It is also the second most commonly impaired domain in MCI, following memory impairment [70]. Individuals with MCI exhibit greater decreases in white matter integrity. This results in a dysexecutive syndrome, which impairs set-shifting and task-switching [71, 72] and affects memory performance and learning [73, 74].

Regarding the second hypothesis, the results confirm the importance of cognitive flexibility, specifically set-shifting, in higher-level executive functioning in older adults with MCI and, to a lesser extent, in healthy older adults. The term “flexibility” is used to describe the ability to adapt new behaviors in response to unexpected environmental demands, which is crucial for the successful development of higher-level executive functions. Individuals with Mild Cognitive Impairment have greater difficulty responding to novel demands and shifting attention from one task to another while perseverative behaviors persist [7].

The results indicate that cognitive flexibility predicts higher executive functioning in both healthy older adults and those with Mild Cognitive Impairment. However, in the control group, cognitive flexibility only predicts fluid intelligence and not planning. In contrast, both linear regression models were significant for older adults with Mild Cognitive Impairment. The variable that significantly influences the models analyzed in the two groups is set-shifting. However, a distinct pattern is observable between the two groups. Cognitive flexibility explains a higher percentage of the variance in fluid intelligence than in planning. Furthermore, cognitive flexibility accounts for a greater proportion of the variance in the group with Mild Cognitive Impairment for both fluid intelligence and planning.

This result can be explained by the more significant structural and functional changes observed in individuals with MCI compared to healthy participants. Specifically, patients with MCI have greater alterations in the frontal and striatal areas responsible for executive functioning [63–67]. On the other hand, physiological aging leads to less severe neurobiological and neuropathological changes than those observed in patients with Mild Cognitive Impairment [75].

## Conclusions

To the best of our knowledge, this study is the first to analyze the components of cognitive flexibility and their role in higher-level executive functioning in Mild Cognitive Impairment. The findings indicate that participants with MCI had a more pronounced decline in executive functioning and that set-shifting can differentially predict planning and fluid intelligence in both MCI and the healthy population. Given that executive functioning is a recognized marker of cognitive impairment and an increased risk of transition from MCI to Alzheimer’s disease [68, 69], this finding has significant implications for the prevention of mild and severe cognitive decline in the elderly. Deficits in executive functioning are linked to decreased functional abilities, reduced independence, and heightened challenges in daily activities [54]. Moreover, they increase the risk of cognitive decline and progression from Mild Cognitive Impairment to dementia [55, 56]. Consequently, early intervention targeting cognitive flexibility, particularly set-shifting, which is subject to a greater physiological decline, has the potential to mitigate the general cognitive decline and facilitate successful aging. In particular, acting on executive functioning could slow the progression of pathological decline, because the dysfunctions of EFs are subject to greater deterioration, specifically in Alzheimer’s disease [76].

### Limits and future directions

Although the results are interesting, the study has some limitations.

The limited sample size constrains the generalizability of our findings and weakens the statistical strength of our analyses, potentially impacting the reliability of our conclusions. Moreover, the study design was cross-sectional, thus precluding the establishment of causality or the observation of changes over time. This design captures data at a single point in time, making it challenging to determine the direction of relationships between variables or assess how variables change over time. Ultimately, relying on a single test to evaluate planning and fluid intelligence could have posed an additional constraint. Higher-order executive functions are extremely complex, and the use of one single test may only partially capture this aspect.

In order to enhance the generalizability of our results and enable further analyses, particularly regarding the differentiation between amnestic and non-amnestic MCI types, we plan to increase the sample size in future studies. Future research could investigate potential variations among MCI subtypes to gain a more nuanced understanding of cognitive impairment in this population. Moreover, we plan to use multiple measures and cognitive tasks to assess higher-order executive functions. This will permit a more comprehensive investigation of these constructs. Finally, longitudinal studies are necessary to address the aforementioned limitations.

## Data Availability

No datasets were generated or analysed during the current study.

## Declarations

The purpose of the study was explained to each participant so that they could voluntarily decide to participate or withdraw their participation. Then, informed consent for participation in the present study was obtained from all participants. All participants in the study were deemed to ethically consent to their participation in the research presented in this manuscript.

## Abbreviations

ADL: Activities of Daily Living
aMCI – md: Amnestic Mild Cognitive Impairment – multiple domain
aMCI: Amnestic Mild Cognitive Impairment
CFA: Confirmatory Factor Analysis
EFs: Executive Functions
gF: Fluid Intelligence
HC: Healthy Control
IADL: Instrumental Activities of Daily Living
MCI: Mild Cognitive Impairment
MMSE: Mini Mental State Examination
naMCI – md: Non-amnestic Mild Cognitive Impairment – multiple domain
naMCI: Non-amnestic Mild Cognitive Impairment
PF: Phonemic Fluency Test
SF: Semantic Fluency Test
TMT: Trail Making Test
ToL: Tower of London
WCST: Wisconsin Card Sorting Test
